# Transcriptome Analysis of Differentially Expressed Genes Induced by Low and High Potassium Levels Provides Insight into Fruit Sugar Metabolism of Pear

**DOI:** 10.3389/fpls.2017.00938

**Published:** 2017-05-31

**Authors:** Changwei Shen, Jie Wang, Xiaoqian Shi, Yalong Kang, Changyan Xie, Lirun Peng, Caixia Dong, Qirong Shen, Yangchun Xu

**Affiliations:** Key Laboratory of Plant Nutrition and Fertilization in Low-Middle Reaches of the Yangtze River, Ministry of Agriculture, Jiangsu Key Laboratory of Solid Organic Waste Utilization, Jiangsu Collaborative Innovation Center for Solid Organic Waste Resource Utilization, College of Resources and Environmental Science, Nanjing Agricultural UniversityNanjing, China

**Keywords:** pear, fruit, sugar metabolism, transcriptome analysis, potassium nutrition, RNA-Seq

## Abstract

Potassium (K) deficiency is a common abiotic stress that can inhibit the growth of fruit and thus reduce crop yields. Little research has been conducted on pear transcriptional changes under low and high K conditions. Here, we performed an experiment with 7-year-old pot-grown “Huangguan” pear trees treated with low, Control or high K levels (0, 0.4, or 0.8 g·K_2_O/kg soil, respectively) during fruit enlargement and mature stages. We identified 36,444 transcripts from leaves and fruit using transcriptome sequencing technology. From 105 days after full blooming (DAB) to 129 DAB, the number of differentially expressed genes (DEGs) in leaves and fruit in response to low K increased, while in response to high K, the number of DEGs in leaves and fruit decreased. We selected 17 of these DEGs for qRT-PCR analysis to confirm the RNA sequencing results. Based on GO enrichment and KEGG pathway analysis, we found that low-K treatment significantly reduced K nutrient and carbohydrate metabolism of the leaves and fruit compared with the Control treatment. During the fruit development stages, *AKT1* (*gene39320*) played an important role on K^+^ transport of the leaves and fruit response to K stress. At maturity, sucrose and acid metabolic pathways were inhibited by low K. The up-regulation of the expression of three *SDH* and two *S6PDH* genes involved in sorbitol metabolism was induced by low K, promoting the fructose accumulation. Simultaneously, higher expression was found for genes encoding amylase under low K, promoting the decomposition of the starch and leading the glucose accumulation. High K could enhance leaf photosynthesis, and improve the distribution of the nutrient and carbohydrate from leaf to fruit. Sugar components of the leaves and fruit under low K were regulated by the expression of genes encoding 8 types of hormone signals and reactive oxygen species (ROS). Our data revealed the gene expression patterns of leaves and fruit in response to different K levels during the middle and late stages of fruit development as well as the molecular mechanism of improvement of fruit sugar levels by K and provided a scientific basis for improving fruit quality with supplemental K fertilizers.

## Introduction

Pear (*Pyrus* L.) is cultivated world-wide, with China ranking as the top producer of Asian pear (Yang et al., 2015). Sugar is one of the most important factors for fruit quality and is the main energy source for fruit metabolism (Zhang et al., 2016). In *Pyrus* species, sucrose and fructose are the major soluble sugars, and overall sugar content varies greatly between different cultivars. But the low sugar content in fresh pear directly influences the sweetness of fruit, leading to great economic losses in postharvest storage periods (Hudina and Śtampar, 2005). Potassium K^+^ is an essential plant nutrient and plays a major role in different physiological processes such as turgor regulation, osmotic adjustment, stomatal movement, cell elongation, and signal transduction (Clarkson and Hanson, 1980; Armengaud et al., 2004). K^+^ contributes to balancing the electrical charge of membranes, movement of ions, enzyme activation, stabilization of protein synthesis, and metabolism of sugars (Maathuis and Sanders, 1996). However, K availability is often limited in natural and agricultural ecosystems. K^+^ deficiency directly affects plant growth, leading to decreased crop yield and production, so supplemental K fertilizers are required for sustainable agricultural practices.

Previous studies have reported the detailed physiological mechanisms of K absorption and uptake in several plant species, and the molecular pathways of transporters and channels involved in the uptake and mobilization of potassium from roots to other organs in *Arabidopsis* have been reported (Véry and Sentenac, 2003; Grabov, 2007; Yao et al., 2010). In *Arabidopsis thaliana*, it showed that more than 30 membrane proteins, which are grouped into five families (Shaker, TPK, Kir-like, HAK/KUP/KT, and KEA), are dedicated to K^+^ transport. The Shaker channel family and the HAK/KUP/KT transporter family play major roles in K^+^ uptake from the soil and in long-distance K^+^ transport in plants. Maathuis and Sanders (1996) observed that low-affinity K^+^ uptake is passive and mediated by channels, whereas high-affinity K^+^ uptake is active and mediated by co-transporters (probably H^+^:K^+^ symporters). *AKT1* and *HAK5* are the main players in K^+^ uptake when the external concentration of this cation is below 100 μM (Rubio et al., 2008). *AKT1* function is activated through phosphorylation, which is mediated by CBL (calcineurin B-like protein)-interacting protein kinases (CIPKs) (Xu et al., 2006). In *Arabidopsis*, the SKOR channel, a member of the Shaker family, is expressed in the root stele, where it contributes to K^+^ secretion into the xylem sap toward the shoots (Gaymard et al., 1998). It is sensitive to the external concentration of K^+^; the likelihood of its opening decreases when the external concentration of K^+^ is increased (Johansson et al., 2006). The regulation of the Shaker channel has been related to the control of membrane transport energization, particularly of sucrose transport into and out of the phloem sap, leading to improved apoplastic sugar allocation (Gajdanowicz et al., 2011). Armengaud et al. (2009) reported that metabolite profiles of low-K *Arabidopsis* plants were characterized by a strong increase in the concentrations of soluble sugars (sucrose and fructose) and these increased significantly within 24 h of K resupply. In comparison with WT rice, *OsHAK16p:WOX11* transgenic lines showed higher soluble sugar concentrations in roots, particularly under low-K conditions. Improvement of sugar partitioning to the roots in the transgenic lines was linked to increased expression of Os*SUT1*and Os*SUT4* in leaf blades and several Os*MSTs* in roots (Chen et al., 2015). Targeted and systematic studies aimed at identifying genes that are up-regulated after a few hours of K^+^ deprivation and/or down-regulated after K^+^ resupply have pinpointed new candidate genes and functions putatively involved in K^+^ signaling. In rice, only a few genes showed changes in expression after this short time, suggesting that most initial events were post-translational (Armengaud et al., 2004; Ma et al., 2012; Takehisa et al., 2013). Many processes and functions are affected by changes in plant K^+^ status, such as metabolic processes (as a long-term consequence of cytoplasmic K^+^ decrease), ROS signaling, and protein phosphorylation/dephosphorylation (by CIPKs and phosphatases, for example) (Ma et al., 2012). The genes related to K^+^ signing transduction, hormone signaling, and ROS signaling have important roles in the regulation of plant defense (Shankar et al., 2013; Fan et al., 2014). However, research related to the molecular mechanisms of K^+^ sensing, uptake, distribution, homeostasis and sugar metabolism in rosaceous fruit trees is still lacking.

Understanding how plants regulate K^+^ transport and sugar metabolism of leaves and fruit to acclimate to low and high K stress and ascertaining the mechanism that K improves fruit quality are valuable. In the past decade, the complete genomes of apple (Velasco et al., 2010), strawberry (Shulaev et al., 2011), plum (Zhang et al., 2012), pear (Wu et al., 2013), and peach (Verde et al., 2013) have been sequenced, making it possible to analyse differentially expressed genes based on the reference genome to reveal different genotypes. RNA-Seq technologies are needed to identify and clarify biosynthetic pathways of secondary metabolites to accelerate metabolomic studies in plants and provide valuable information for identifying differentially expressed genes during a developmental stage or in response to a particular physiological condition. Although some transcriptomic studies of fruit trees have focused on the roles of various stresses, such as cold (Xin et al., 2013), heat (Rocheta et al., 2013, drought (Corso et al., 2015), salt (Daldoul et al., 2010), and high light (Carvalho et al., 2011), in fruit development, no information is available on the study of response mechanism of pear leaves and fruit in suffering from K^+^ stress.

In our previous research, we found that pear fruit size sharply increased from the enlargement stage to maturity (Shen et al., 2016). A large number of mineral elements and carbohydrates are needed in fruits in the later stages of their development (data unpublished). During the grape berries development, cell expansion at veraison phase is driven by an increase in sugar in the cell vacuole, and K plays an important role in the accumulation of sugars (Davies et al., 2006). In this study, “Huangguan” pear (*Pyrus bretschneideri* Rehd. cv. Huangguan), the major variety planted in Hebei, Shandong, Jiangsu and Gansu Provinces in China, was used as an experimental subject. RNA-Seq technology was applied on the leaves and fruit at enlargement II and maturity stages to reveal the molecular mechanism of sugar metabolism affected by different K levels. Some important candidate genes associated with K transport and sugar metabolism and signaling pathways were identified to provide a valuable resource to further understand the major transcriptomic regulatory pathways in response to different K levels (Low K and High K) in pear leaves and fruit.

## Materials and methods

### Plant materials and K^+^ treatments

The experiment was carried out with 7-year-old “Huangguan” pear trees using pot (diameter 42 cm; height 42 cm) culture in 2016. The pear trees were cultivated under natural conditions at Pailou Teaching and Research Farm of Nanjing Agricultural University. Each pot was filled with 25 kg of soil mix (clay soil/sand, 80:20 ratio, w/w). Urea (46.4% N), phosphate (12% P_2_O_5_) and potassium oxide (51% K_2_O) fertilizers were used as the sources of N, P, and K, respectively. All pots had the same N (0.4 g/kg soil) and P_2_O_5_ (0.2 g/kg soil) levels; the experiment was set up using the following three K_2_O levels: Low K, Control and High K [0 (K0), 0.4 (K0.4), and 0.8 (K0.8) g kg^−1^ soil, respectively]. Each K treatment was replicated three times in a completely randomized design, with five trees in each replication. Each tree around the drip line in the orchard was supplied with urea (46% N) (base fertilizer period accounted for 40% in 2015, budding period accounted for 40% in 2016, enlargement stage I accounted for 20%), calcium superphosphate (12% P_2_O_5_) at the base fertilizer period, and potassium sulfate (51% K_2_O) (base fertilizer period accounted for 20% in 2015, budding period accounted for 40%, enlargement stage I accounted for 40%). The fertilizers were first dissolved in 1 L of water and then used for drip irrigation. Samples were collected at six developmental stages from the day after full blooming (0 DAB, S0). Leaves and fruit were selected at the physiological fruit dropping stage (20 DAB, S1), the young fruit stage (50 DAB, S2), the fruit rapid enlargement I stage (80 DAB, S3), the fruit rapid enlargement II stage (105 DAB, S4) and the mature stage (129 DAB, S5). To the sampling stage of 80 DAB, samples were first collected and then the plant was applied by K fertilizer. According to the dynamic change of fruit growth (including the fruit weight and diameter, see Figure S1) and the concentration of four sugar components (fructose, sorbitol, glucose and sucrose) (data unpublished), the enlargement II and the maturity stages were found to be the vital stages determining for fruit quality. So, only these two stages were selected to study the transcriptome (with three biological replicates). The sampled fruit and leaves were weighed, immediately frozen in liquid N2, and stored at −80°C for sugar measurement and RNA-Seq analysis.

### Measurement of leaf photosynthetic parameters

A LI-6400 portable photosynthesis system (Li-Cor, Lincoln, NE, USA) was used for *in vivo* measurements of photosynthetic parameters, including the net photosynthetic rate (Pn), stomatal conductance (Gs), intercellular CO_2_ concentration (Ci), and transpiration rate (Tr) on sunny days between 9:00 and 11:00 a.m. after 80 DAB in the summer. Leaves were illuminated with a 6400-02B light source at a saturating incident photosynthetic photon flux density of 1,000 μmol m^−2^ s^−1^.

### Analysis of individual carbohydrates in leaves and fruit

High-performance liquid chromatography (HPLC) was used to analyse the carbohydrates present in pear fruit. The method used to extract the sugar from the leaves and fruit flesh was slightly modified from Melgarejo et al. (2000). Pear leaf and fruit samples (2 g) were homogenized to a purée and diluted to 20 mL with distilled water. The extract was then centrifuged at 12,000 × g for 15 min. The supernatant was filtered three times through a 0.45-μm membrane filter (Waters, Milford, MA). The optimal HPLC conditions to measure sugar components were as follows (Karkacier et al., 2003): CAPCELL PAK NH2 (4.6 × 250 mm, 5 μm) column (Shiseido, Tokyo, Japan), constant temperature of 50°C, and CH_3_CN and H_2_O at a volume ratio of 80:20. An evaporative light scattering detector (ELSD) (Alltech 3300 ELSD; Grace, Deerfield, IL) was used with a flow rate of 1.0 mL min^−1^; the drift tube temperature was 80°C and the nitrogen flow rate was 2.0 mL min^−1^. The injection volume was 5 μL. Standard curves for sorbitol, fructose, glucose, and sucrose (Sigma, St Louis, MO) were generated as references to quantify the sugar concentration in the samples. Total sugars were calculated as the summed concentrations of individual sugars.

### Measurement of K concentrations in leaves and fruit

Leaves were rinsed twice with tap water and twice with deionized water, wiped with a paper towel and thoroughly dried. After weighing, one quarter of the fruit were randomly selected, homogenized and thoroughly dried. All plants were killed by heating at 105°C and were then dried in an oven at 75°C for 2 d. Dried leaves and fruit were ground, and the K concentrations of leaf and fruit samples of ~0.500–1.000 g were determined by Inductively Coupled Plasma-Atomic Emission Spectrometer (ICP-AES, LEEMAN, Delaware, USA) using HNO_3_-HClO_4_ digestion solution (3:1 V:V).

### RNA extraction, library preparation, and RNA-Seq

Because two stages (105 DAB and 129 DAB) are important for pear fruit cell enlargement and ripening, we collected leaf and fruit tissue samples at both of these stages and extracted total RNA using a modified CTAB method (Zhang et al., 2014). RNA-Seq was used to profile transcriptomes for 12 stage, tissue and treatment combinations, each with three biological replicates. Total RNA samples were sent to Vazyme Biotech Co. Ltd. (China) for library construction, sequencing, data pre-processing and gene mapping. To eliminate genomic DNA contamination, the RNA samples were treated with RNase-free DNase I (Takara, Tokyo, Japan). The quality of the RNA was checked using an Agilent 2100 RNA Bioanalyzer (Agilent, Santa Clara, USA). VAHTS mRNA-Seq v2 Library Prep Kit (Vazyme Biotech Co., Ltd. Nanjing, China) was found to meet the requirements for RNA-Seq library construction. Sequencing was performed using a HiSeqXTen sequencing system (Illumina, Inc., San Diego, CA, USA). In addition, the sequencing data were submitted to the National Center for Biotechnology Information Sequence Read Archive (SRA) under accession number SRP099937 (https://trace.ncbi.nlm.nih.gov/Traces/sra/).

### RNA-Seq read processing, assembly, and transcriptome sequence analysis

The resulting libraries were sequenced on an Illumina (HiSeqXTen) using a standard pair-end 150 bp sequencing protocol. The original image data obtained by high-throughput sequencing was converted into sequence data by CASAVA base calling. Each sample generated more than 6 gigabytes of data. For further analysis, the reads were filtered from raw sequencing data with adaptor removed and low-quality (<Q30) reads trimmed. Then the processed reads were mapped to the reference genome “Dangshansuli” (*Pyrus bretschneideri*) (http://www.ncbi.nlm.nih.gov/genome/?term=Pyrus%20x%20bretschneideri) by Tophat2 (Kim et al., 2013) program using the following parameters: segment length, 25; segment mismatches, 2. For the remaining parameters, the default settings were used. The uniformity, insert length, and saturation of sequencing data were analyzed based on the alignment results.

The quantitative analysis of gene expression was completed by Cufflinks based on RPKM (reads per kb per million reads). Differential expressed genes (DEGs) were identified with adjusted *P*-value < 0.05 for multiple tests by Benjamini-Hochberg method (1995) for controlling false discovery rate. Cluster analysis was performed on differentially expressed genes using software cluster and Java Treeview. Gene functional annotations were based on the pear genome database and mapped onto GO terms. GO enrichment analysis was carried out using WEGO. Kyoto Encyclopedia of Genes and Genomes (KEGG) pathways were identified according to *p-*values and adjusted *q*-values using a BLAST search against the KEGG database and were then mapped onto KEGG pathways (Young et al., 2010).

### Quantitative RT-PCR analysis

Based on the target gene sequences, 17 gene-specific primer pairs were designed (Table S1). The RNA samples used for quantitative real-time polymerase chain reaction (qRT-PCR) analysis were aliquots of the samples used in the RNA-seq experiments. cDNA was synthesized using 1 μg of total RNA with the Primer Script RT Reagent Kit (Takara, Dalian, China) in a 20-μl reaction volume. Three biological replicates were performed in each experiment. The total reaction volume for each qRT-PCR was 20 μL, which comprised 10 μL SYBR Green PCR SuperMix (Vazyme Biotech Co., Ltd. Nanjing, China), 0.4 μL of each primer, 2 μL of 1:10 diluted cDNA, 0.4 μL passive reference dye and 6.8 μL double-distilled water. The PCR reaction conditions were as follows: 94°C for 4 min and 40 cycles of 94°C for 5 s followed by 60°C for 30 s. Reactions were performed using an Applied Biosystems 7500 Real-Time PCR system. The pear β*-TUB* gene was used as a control for normalization of expression, and relative expression was calculated by the 2^−ΔΔCt^ method (Livak and Schmittgen, 2001).

### Statistical analysis

All statistical analyses were performed with SPSS 18.0 (SPSS Inc., Chicago, IL, USA) and Microsoft Excel (Microsoft Corporation, Redmond, WA, USA). Data were analyzed by one-way analysis of variance (ANOVA). Mean separations were performed by Duncan's multiple range tests. OriginPro 8.1 (Origin Inc., Chicago, IL, USA) was used to draw the figures. Differences at *P* < 0.05 were considered significant.

## Results

### Effect of different K levels on potassium nutrient levels and leaf photosynthetic characteristics

**Figure 1:**
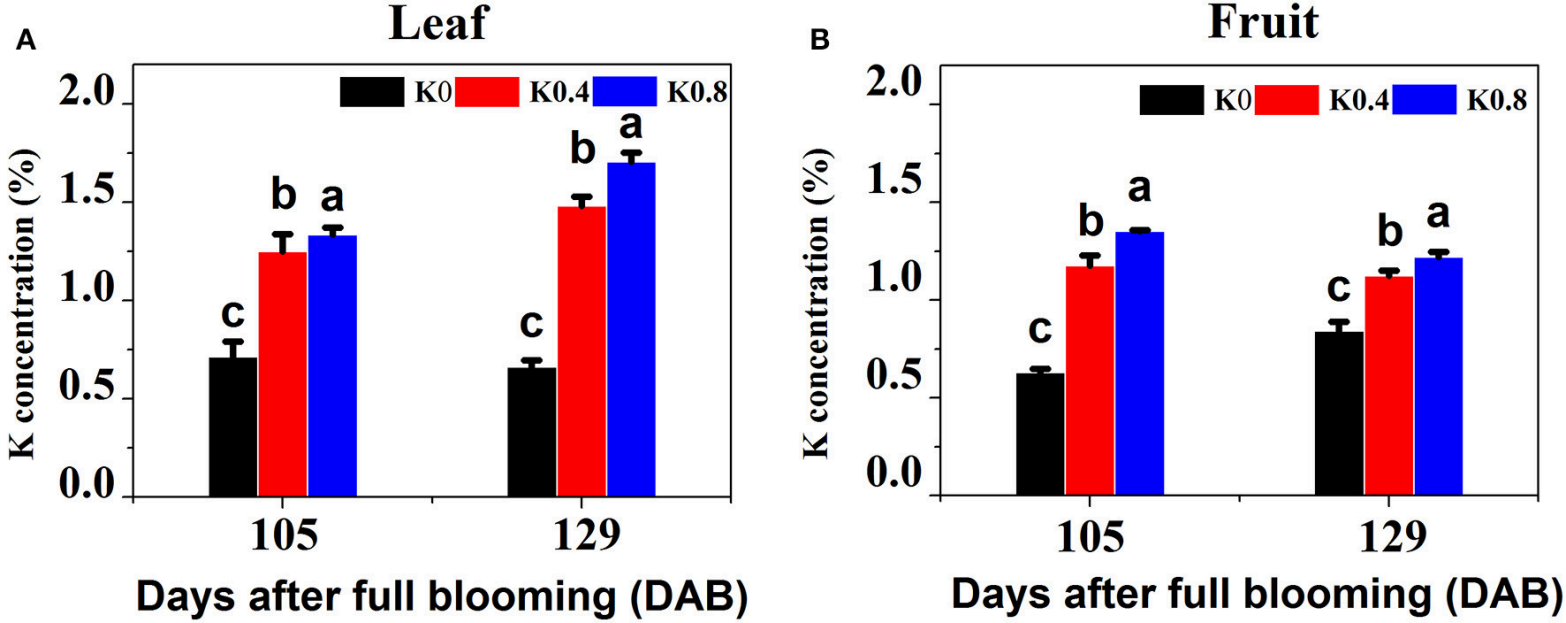
Effect of different potassium (K) levels on K concentration of the leaves and fruit of “Huangguan” pear at 105 and 129 DAB. Each point represents the average of six samples. Error bars represent SE. Statistical analysis was performed by LSD test at *P* < 0.05.

To determine which stages of fruit development could be used for profiling transcriptomes related to sugar metabolism in pear leaves and fruit, we tracked and analyzed the six processes of fruit development from flowering (0 DAB) to maturity (129 DAB), and determined single-fruit weight (Figure S1B) and fruit diameter (Figure S1C) under the different K treatments. We found that after 80 DAB, the single-fruit weight and the fruit diameter rapidly increased, and the average weight at 105 DAB was 3.38 times that at 80 DAB. All fertilizer application was completed at 105 DAB, which was a key stage for rapid enlargement of fruit volume and large demand for nutrients. The aim was to elucidate the effect of K on the absorption of potassium in leaves and fruit and the changes of fruit sugar concentration during fruit development and maturation, so we focused on the leaves and fruit at 105 and 129 DAB. We found that K concentrations in leaves and fruit under the different K treatments were significantly different at 105 DAB. Leaf K concentrations under the K0.4 and K0.8 treatments were significantly higher 106.94 and 127.05% than that under the K0 treatment in leaves, respectively. Fruit K concentrations under the K0.4 and K0.8 treatments were significantly increased 86.90 and 114.96, respectively, compared to K0 treatments in fruit. At maturity (129 DAB), leaf K concentrations under the K0.4 and K0.8 treatments were significantly higher than that under the K0 treatment (75.43 and 87.26%, respectively). The K concentrations in fruit under the K0.4 and K0.8 treatments were elevated by 32.33 and 43.86%, respectively (Figure 1).

**Figure 2:**
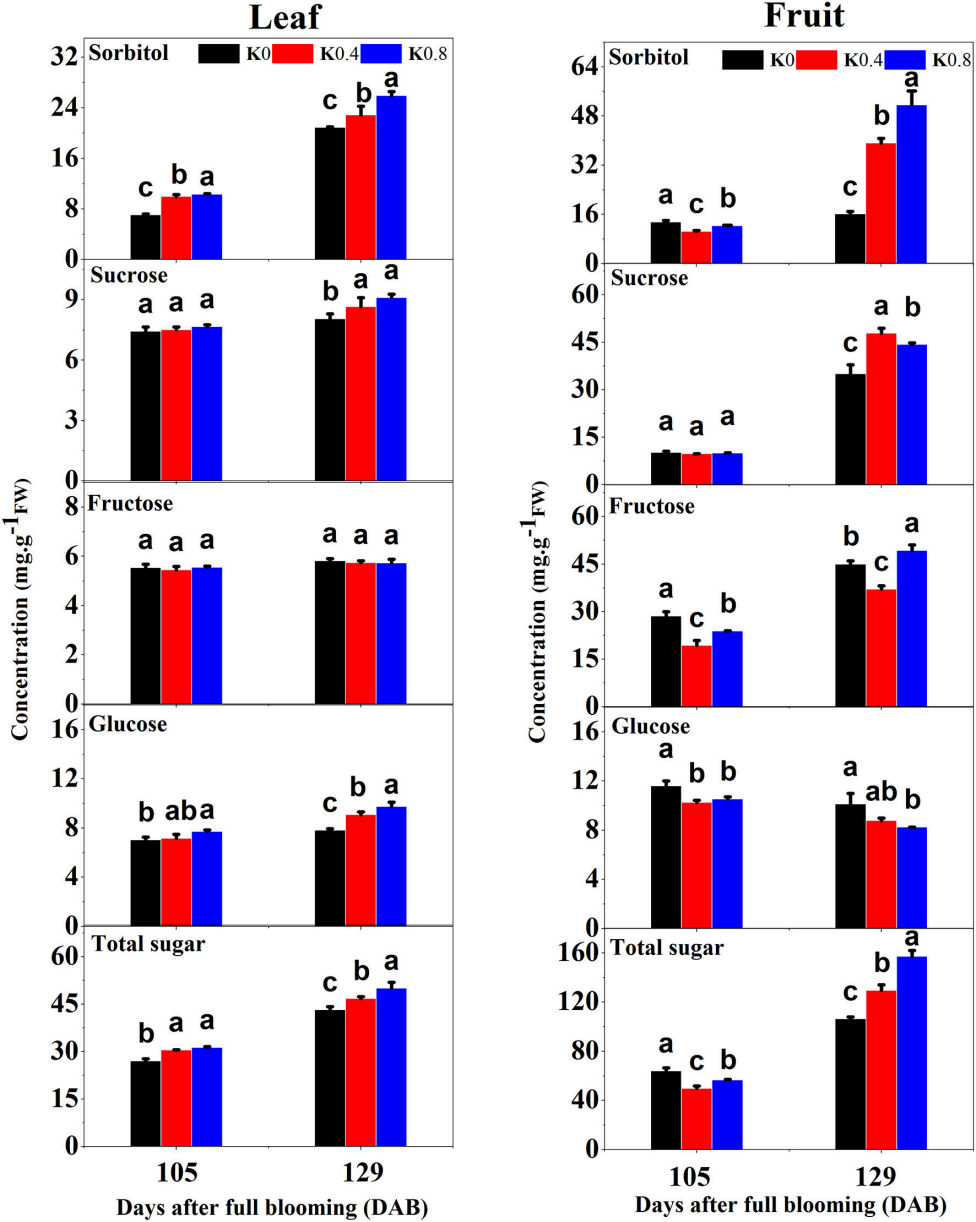
Effect of different potassium (K) levels on sorbitol, sucrose, fructose, glucose, and total sugar concentration of the leaves and fruit of “Huangguan” pear at 105 and 129 DAB. Each point represents the average of six samples. Error bars represent SE. Statistical analysis was performed by LSD test at *P* < 0.05.

This study found that the concentrations of sorbitol, glucose and total sugar in leaves at 105 DAB increased with increasing K application rates. The concentrations of sorbitol, fructose, glucose and total sugar under the K0 treatment in fruit were significantly greater than those in the K0.4 and K0.8 treatments. At 129 DAB, the concentrations of sorbitol, sucrose and glucose in leaves under the K0.4 and K0.8 treatments were significantly higher than that under the K0 treatment. The concentrations of sorbitol and sucrose in fruit under the K0.4 and K0.8 treatments were significantly higher than that of K0 treatment, while the concentrations of fructose and glucose under the K0 treatment was significantly higher than that of the K0.4 treatment. In general, the total sugar concentrations in leaves and fruit increased with increasing K application rates at the mature stage (Figure 2). K increases the photosynthetic and CO_2_ assimilation rates of crop leaves and facilitates carbon movement (Hu et al., 2016). In this study, the photosynthetic characteristics of leaves under different K levels were measured before the fruit rapid enlargement II stage. We found that the net photosynthetic rate (Pn), stomatal conductance (Gs) and transpiration rate (Tr) increased gradually with increasing K application rates, while the intercellular CO_2_ concentration (Ci) decreased (Figure S2).

### RNA-Seq libraries of leaves and fruit

Paired-end RNA-Seq reads were generated with a length of 150 bp. Mapping the sequence reads to the pear genome revealed that a total of 46.84 million (leaf) and 55.30 million (fruit) reads were generated. After removing adapters, low-quality regions, and possible contamination, we obtained more than six clean gigabases with a GC percentage above 47.24% and a Q30 percentage above 95.36%. The proportion of clean reads in the pear transcriptome libraries that mapped to the pear reference genome ranged from 76.6 to 80.9% (except for L0S5_2), while the adjusted proportion ranged from 70.0 to 72.9% (Table S2). Mapping of exons, introns and intergenic regions to the pear reference genome accounted for an average of 95.72, 2.32, and 1.96%, respectively (Table S3).

### Gene expression profiles of leaves and fruit at 105 and 129 Dab in response to low and high K

**Figure 3:**
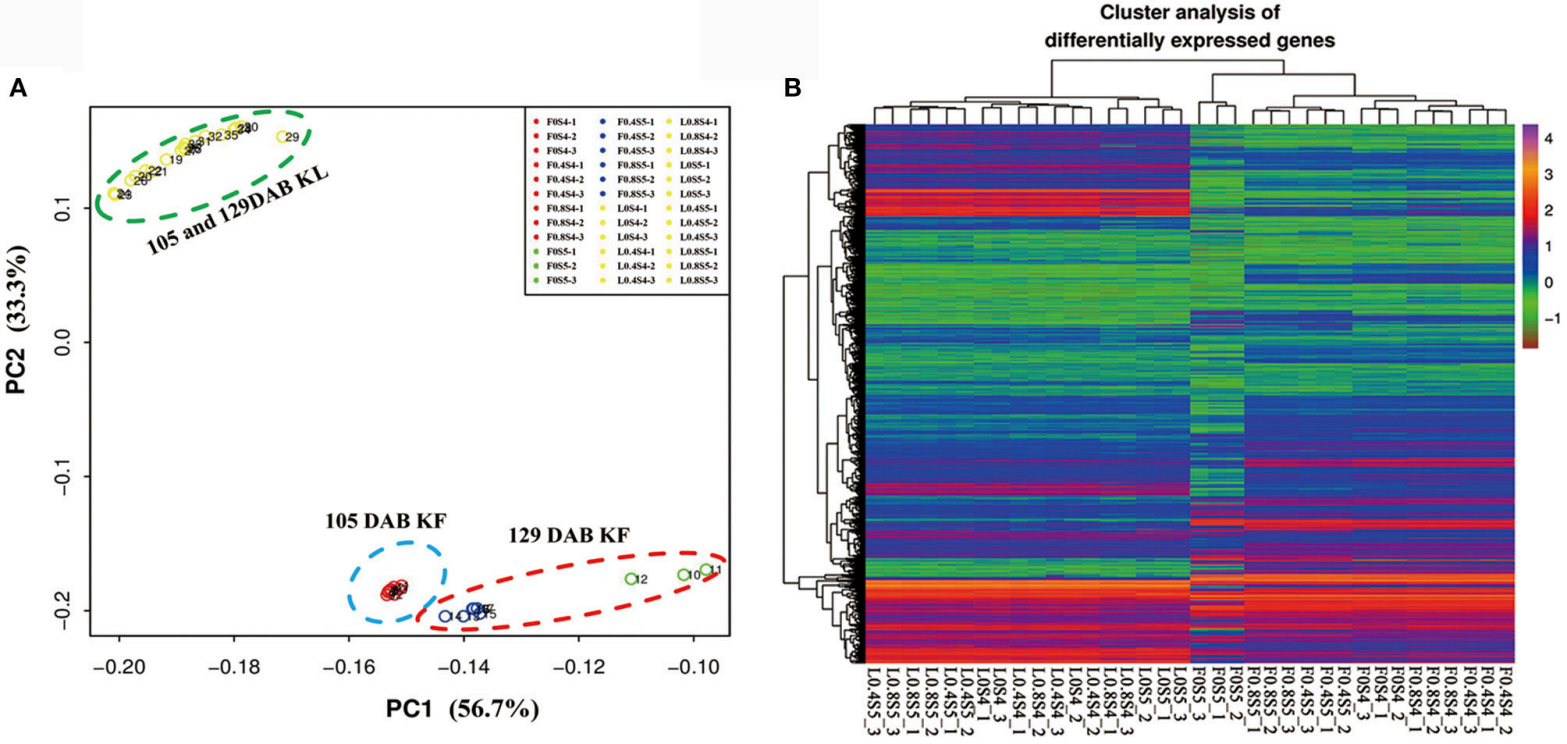
Principal component analysis of all leaf and fruit transcripts, and cluster analysis of the differentially expressed genes. **(A)** Principal component analysis plot of RNA-Seq data. Expression of 36,444 genes in each biological replicate for every K treatment at 105 and 129 DAB (days after full blooming) were used for principal component analysis. KL: K treatments in leaf; KF: K treatments in fruit. **(B)** Heatmap of 36,444 differentially expressed genes in leaf and fruit between 105 and 129 DAB (days after full blooming).

Mapping the sequence reads to the pear genome revealed that a total of 36,444 unique genes had perfect matches with pear genes. K-means clustering via principal component analysis of these genes from 36 samples in leaves and fruit separated the genes into two groups. 18 leaf samples (105 DAB and 129 DAB) had homogenous gene expression patterns, while gene expression patterns of 18 fruit samples were clustered into three groups: nine samples under different K levels at 105 DAB, three samples under K0 treatment at 129 DAB, and six samples under K0.4 and K0.8 treatments at 129 DAB (Figure 3A). Hierarchical clustering of 36,444 differentially regulated genes (Figure 3B) exhibited similar expression patterns in the principal component analysis. We found that expression profiles of 18 leaf samples did differentiate, while expression profiles of 18 fruit samples clustered together. Moreover, 3 replicate samples under K0 treatment in fruit at 129 DAB clustered together, with other samples being significantly different. Overall, these evaluations indicate that our samples in this study were reproducible.

### Identification and qRT-PCR analysis of the DEGs of leaves and fruit at 105 and 129 Dab in response to low and high K

**Figure 4:**
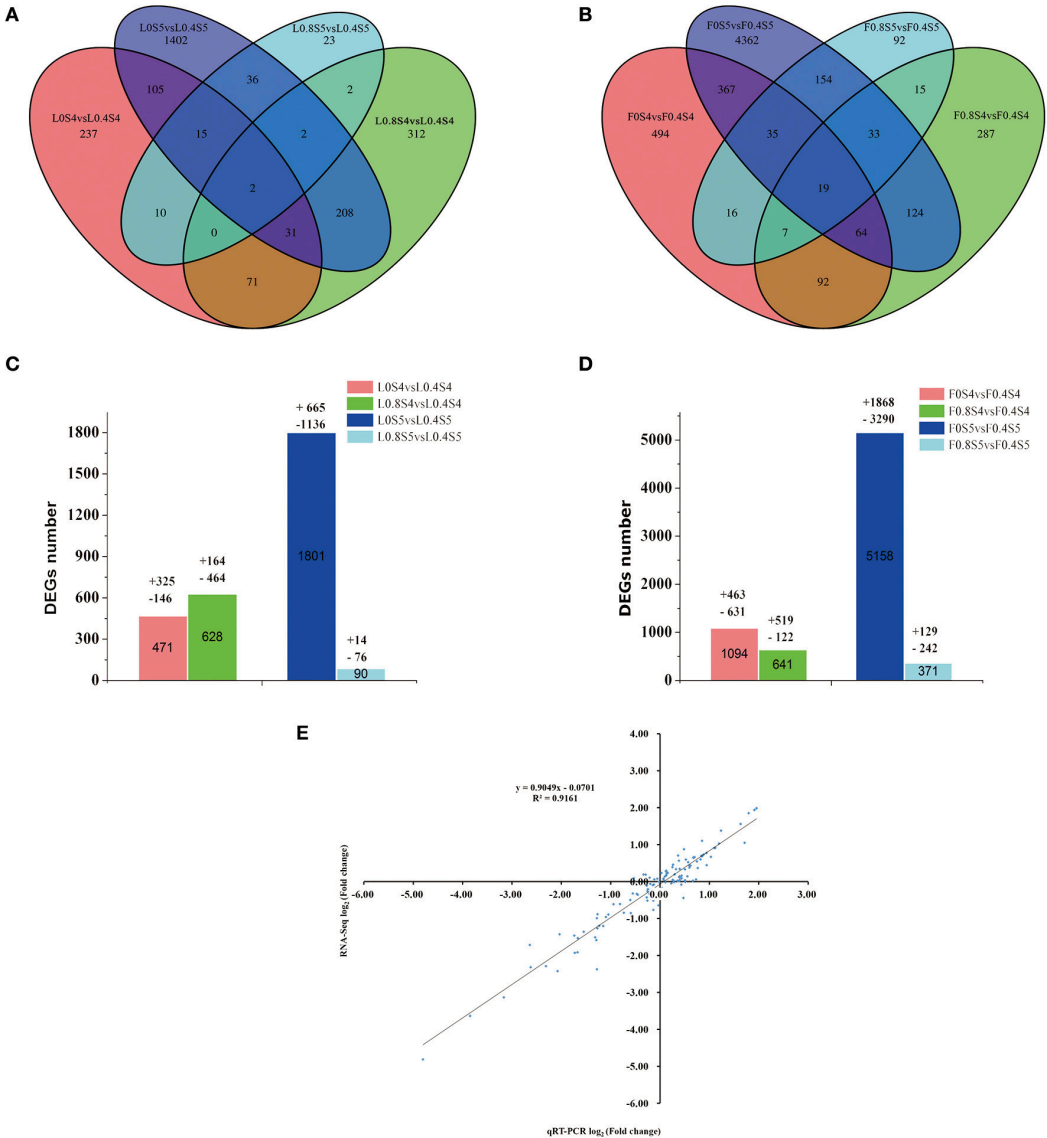
Venn diagram of the differentially expressed genes (DEGs) of RNA-Seq (y axis) and qRT-PCR data (x axis). **(A,B)** Venn diagram showing the number of differentially expressed genes (*P* < 0.05 and fold change equal or greater than 2) from 105 to 129 DAB under different K levels. **(C,D)** The total number of DEGs, up-regulated and down-regulated, in leaf and fruit between 105 days and 129 DAB. **(E)** A qRT-PCR assay was carried out for 17 randomly selected DEGs. Values are the log_2_ ratio (low K/Control or high K/Control) for genes. The correlation coefficient (*R*^2^) is indicated in the figure.

As shown in Figures 4A,B, compared to the Control treatment (L0.4S4), a total of 471 genes were differentially expressed under low-K (L0S4) treatment in leaves at 105 DAB, with 325 up-regulated and 146 down-regulated. A total of 628 genes were differentially expressed under high-K (L0.8S4) treatment in leaves, with 164 up-regulated and 464 down-regulated. At 129 DAB, compared to the Control treatment (L0.4S5), a total of 1801 genes were differentially expressed under low-K (L0S5) treatment in leaves, with 665 up-regulated and 1,136 down-regulated. A total of 90 genes were only differentially expressed under high-K (L0.8S5) treatment in leaves, with 14 up-regulated and 76 down-regulated (Figures 4A,C). The number of DEGs between the leaves and fruit during the two stages displayed basically the same trend, but compared to the Control treatment (F0.4S4), the number of DEGs under low-K (F0S4) treatment was much greater than that under high-K (F0.8S4) treatment. There were 1,094 genes (463 up-regulated and 631 down-regulated) significantly differentially expressed in the low-K (F0S4) treatment, while only 641 (519 up-regulated and 122 down-regulated) genes were significantly differentially expressed in the high-K (F0S4) treatment. At 129 DAB, the numbers of DEGs between the leaves and fruit in response to low-K (F0S5) and high-K (F0.8S5) treatment were greater than that under Control (F0.4S5) treatment. 1,868 and 129 genes were significantly up-regulated in response to low K and high K, respectively, and 3,290 and 242 genes were down-regulated in response to low K and high K, respectively (Figures 4B,D). Overall, 2 and 19 DEGs in leaves and fruit, respectively, were common in response to both treatment and stage comparisons (Figures 4A,B).

To validate the reliability of the expression profiles obtained from RNA-Seq, we randomly selected 17 DEGs with various degrees of expression for qRT-PCR. The pear β*-TUB* gene was used as a control for expression normalization. These unigenes involved in plant potassium transport, TFs, and the pathways of sugar metabolism and hormone biosynthesis (Table S1). The results showed that although the exact fold change for the selected genes at 105 and 129DAB in leaf and fruit varied between digital gene expression and qRT-PCR analysis, the trend of gene expression changes detected by the two different approaches were largely consistent (Figure S3). Pearson's correlation coefficients showed that qRT-PCR data for these genes and RNA-Seq results were highly correlated (Figure 4E). The correlation coefficient was 0.9161, indicating a positive correlation between RNA-Seq data and qRT-PCR data. This indicates that the RNA-Seq data here are valuable.

### GO analysis of the DEGs of leaves and fruit at 105 and 129 Dab in response to low and high K

To analyse the functions of the genes differentially expressed in response to low and high K between leaves and fruit at 105 and 129 DAB, gene ontology (GO) enrichment analysis was performed on eight sets of DEGs (L0S4 vs. L0.4S4, L0.8S4 vs. L0.4S4, L0S5 vs. L0.4S5, L0.8S5 vs. L0.4S5, F0S4 vs. F0.4S4, F0.8S4 vs. F0.4S4, F0S5 vs. F0.4S5, and F0.8S5 vs. F0.4S5) by singular enrichment analysis (SEA) in the AgriGO database (http://bioinfo.cau.edu.cn/agriGO/index.php) and the session ID of each SEA was recorded. These GO terms were listed with the lowest *P*-value and an adjusted *q*-value ≤ 0.05 (Table S4). We found that the varieties and quantities of GO annotations of DEGs in leaves and fruit at 129 DAB were greatest under low-K treatment, while the varieties and quantities of GO annotations of DEGs were the smallest under high-K treatment. The number of GO terms of DEGs related to cellular component under low-K treatment at 129 DAB in fruit was greater than that in response to low K and high K at 105 DAB; a similar trend was observed in leaves. The GO term related to molecular function in fruit responses to low K was significantly enriched in “catalytic activity” and “oxidoreductase activity.” In biological processes, the GO terms of each group were highly enriched in “response to stimulus,” “single-organism metabolic process” and “response to oxygen-containing compound.” The number of GO terms related to “response to chemical” and “response to stress” in leaves in response to low K was higher and increased with fruit development. A high number of GO terms in fruit in response to low K and high K were also significantly enriched in “response to chemical” and “response to stress.” With fruit ripening, more GO terms related to “oxidation-reduction process” were significantly enriched in fruit. In particular, a high number of GO terms were related to “carbohydrate metabolic process” in low-K fruit at 129 DAB (Table S4).

### KEGG analysis of the DEGs of leaves and fruit at 105 and 129 DAB in response to low and high K

Pathway enrichment analysis of the 8 groups of DEGs using KEGG (http://www.genome.jp/kegg/) identified significantly enriched “metabolic pathways,” “biosynthesis of secondary metabolites” and “plant hormone signal transduction” by comparing these genes with the whole genomic background (Table S5). The KEGG pathways in leaves under low K were also enriched in “plant-pathogen interaction.” The numbers of KEGG pathways and genes in response to low and high K in fruit between 105 and 129 DAB were higher than those in leaves. Over time, the number of pathway species and genes in leaves under low K increased, but that under high K decreased. The number of pathway species and genes in fruit exposed to different K levels also increased. The number of pathway species in response to low K in fruit at 129 DAB exceeded 25; these included significant enrichment in pathways related to sugar metabolism, including “starch and sucrose metabolism,” “galactose metabolism,” “fructose and mannose metabolism” and “nitrogen metabolism.” While no “sorbitol metabolism” pathway was found, the functional annotation of this pathway may not have been released.

### DEGs involved in K^+^ transport

The GO term and KEGG pathway enrichment analyses highlighted the following functional categories: genes related to transmembrane transport, genes related to metabolic pathway (sugar metabolism), genes related to hormone signal transduction, and genes related to reactive oxygen species. Therefore, genes in these categories were investigated in detail. The genes related to potassium transport in leaves and fruit exposed to different K levels at 105 and 129 DAB are listed in Table 1. We found that the number of genes related to potassium transport in fruit under low and high K was greater than that in leaves. These genes could be divided into 4 categories: cation /H^+^ antiporter, K^+^ efflux antiporter, potassium channel proteins and potassium transporters. One and three genes were obviously down-regulated under low K in leaves at 105 and 129 DAB, respectively. Surprisingly, there was no difference under high K. In fruit, only one gene (*gene36660*) and two genes were down-regulated and up-regulated, respectively, in low K at 105 DAB, while 15 genes were down-regulated in high K at 129 DAB. The expression of K transporter (*gene31518*) and K channel *AKT1* (*gene39320*) genes in fruit were up-regulated in response to high K between 105 DAB and 129 DAB.

**Table 1:** List of the differentially expressed genes involved in potassium transporters and channels in response to low K and high K.

**Gene Id**	**LOC ID**	**Log2 (fold change)**								**Annotation**
		**Leaf**				**Fruit**				
		**105 DAB**		**129 DAB**		**105 DAB**		**129 DAB**		
		**K0 vs. K0.4**	**K0.8 vs. K0.4**	**K0 vs. K0.4**	**K0.8 vs. K0.4**	**K0 vs. K0.4**	**K0.8 vs. K0.4**	**K0 vs. K0.4**	**K0.8 vs. K0.4**	
	Up	–	–	–	–	2	1	–	1	
	Down	1	–	3	–	1	–	15	–	
*gene27934*	LOC103927292			–1.33						Cation/H^+^ antiporter 18
*gene23076*	LOC103963568							−1.72		Cation/H^+^ antiporter 20-like
*gene31372*	LOC103931080	−1.06								Cation/H^+^ antiporter 20-like
*gene31371*	LOC103931079							−1.61		Cation/H^+^ antiporter 20-like
*gene36660*	LOC103936860					−1.06				K^+^ efflux antiporter 5-like
*gene10388*	LOC103949642							−2.32		K^+^ efflux antiporter 6-like
*gene1309*	LOC103951984							−1.26		K^+^ efflux antiporter 6-like
*gene17083*	LOC103957005					1.28				Sodium/potassium/calcium exchanger 1-like
*gene39320*	LOC103939805			−1.51				−2.32	1.25	Potassium channel AKT1
*gene11877*	LOC103951275							−1.17		Potassium channel KAT1-like
*gene19309*	LOC103959456					1.64				Potassium channel SKOR
*gene26581*	LOC103967420							−3.19		Potassium channel SKOR-like
*gene6330*	LOC103945167							−1.25		Potassium transporter 10-like
*gene18908*	LOC103958999			−1.50						Potassium transporter 1-like
*gene31518*	LOC103931242						1.61			Potassium transporter 1-like
*gene14129*	LOC103953748							−1.25		Potassium transporter 2-like
*gene23394*	LOC103963935							−1.52		Potassium transporter 8-like
*gene13909*	LOC103953509							−2.96		Putative potassium transporter 12
*gene7303*	LOC103946258							−1.25		Two-pore potassium channel 1-like
*gene24765*	LOC103965433							−1.53		Two-pore potassium channel 1-like
*gene24764*	LOC103965434							−1.03		Two-pore potassium channel 1-like
*gene33628*	LOC103933552							−4.93		Two-pore potassium channel 5-like

### DEGs involved in carbohydrate metabolism (sugar and acid metabolism)

Fruit flavor is highly dependent on the balance of soluble sugars and organic acids in fruit, and the sugar/acid ratio is a large component of fruit flavor. Sugars provide not only energy for metabolism but also signaling molecules for plants. The genes involved in metabolic process in leaves and fruit in response to low and high K were divided into two categories, sugar metabolism and acid metabolism (Table S6). The DEGs involved in sugar metabolism were divided into sucrose metabolism, sorbitol metabolism, starch metabolism and other monosaccharide transporters. During fruit development and ripening, the number of DEGs in leaves involved in sugar metabolism in response to low K at 129 DAB was greater than that at 105 DAB. There were 7 genes (3 up-regulated and 4 down-regulated) involved in sorbitol metabolism that were significantly differentially expressed in low K at 129 DAB, and there was no difference in response to high K in leaves. We found that there were 19 genes (8 up-regulated and 11 down-regulated) involved in sucrose metabolism, and 12 genes (8 up-regulated and 4 down-regulated) and 23 genes (10 up-regulated and 13 down-regulated) were significantly differentially expressed in fruit under low K at 129 DAB. Only 4 genes involved in sugar metabolism in fruit were significantly down-regulated under high K. In acid metabolism, a total of 8 genes, including 1 genes up-regulated and 1 genes down-regulated, were differentially expressed in response to low K in leaves at 105 DAB, and 2 genes were up-regulated and 4 genes down-regulated in response to low K in fruit at 129 DAB (Table S6).

### DEGs involved in hormone signaling

Our results showed that K was closely related to hormone signaling by KEGG pathway analysis. DEGs encoding components of hormone metabolism were clustered into eight groups during low- and high-K treatments in leaves and fruit at 105 and 129 DAB, including auxin signaling (51), cytokinin signaling (32), gibberellin signaling (36), abscisic acid signaling (39), ethylene signaling (31), brassinosteroid signaling (124), jasmonic acid signaling (39), and salicylic acid signaling (21) (Table S7). We found that the numbers of brassinosteroid signaling genes involved in cell division and enlargement and ethylene signaling genes involved in fruit ripening and senescence exceeded the numbers of genes involved in other signaling. Overall, the numbers of hormone signaling DEGs in response to low and high K in fruit were greater than those in leaves. At 105 DAB, the numbers of hormone signaling DEGs in response to low K in fruit were greater than that in response to high K. The DEGs encoding brassinosteroid signaling in leaves were induced by exposure to low K at 129 DAB; this including up-regulated (7) and down-regulated (39) genes, in addition to up-regulated (16), and down-regulated (53) genes in fruit. The DEGs encoding brassinosteroid signaling were induced by exposure to high K at 129 DAB; this included 2 down-regulated genes in leaves and 1 up-regulated gene in fruit (Table S7). There was a difference for the effects of potassium application on key genes involved in ethylene metabolism at different stages and in different organs. We found 1 up-regulated gene and 10 down-regulated genes in response to low K in leaves; in contrast, in fruit, there were 9 up-regulated genes and 5 down-regulated genes differentially expressed under low K.

### DEGs involved with reactive oxygen species

Reactive oxygen species (ROS) are known to be involved in signaling pathways specific to low-K stress conditions. Peroxidase, cytochrome P450s and glutathione S-transferase (GST) play important roles during oxidative stress in plants. Our results showed that a total of 89 genes related to ROS were differentially expressed under low and high K between leaves and fruit, including peroxidase (17), cytochrome P450 (63) and GST (9) genes. In leaves, the number of genes encoding cytochrome P450s under low K at 129 DAB was greater than that at 105 DAB. These included 4 up-regulated and 18 down-regulated genes. In fruit, the number of genes encoding cytochrome P450s under low K at 129 DAB was greater than that at 105 DAB, including 13 up-regulated and 26 down-regulated genes (Table S8).

## Discussion

### *AKT1* gene plays a key role in the K transport and assimilation in leaves and fruit

As it is known that K deficiency can influence the uptake and accumulation of this nutrient and its corresponding transporters (Armengaud et al., 2004; Ma et al., 2012), it is not surprising that expression of many K transporters in pear leaves and fruit were also affected under low and high K. The experimental results indicated that the concentrations of K in pear leaves and fruit were significantly lower under low K treatment than that under Control treatment at 105 and 129 DAB (Figure 1). This showed that pear vegetative and reproductive growth were poor during K deprivation, and the K transportation from pear root to shoot may have been inhibited under low K. Ma et al. (2012) reported that the K concentrations of rice roots and shoots under low K stress were lower than that under normal K condition, and the express levels of three *OsHAK* genes (1, 7, and 11) were markedly up-regulated under low-K stress. Unlike that study, our research focused on shoots (the leaves and fruit). This study found that compared to the Control treatment, the gene expression levels of K transporters and channels were significantly down-regulated under low K treatment in leaves and fruit, and the number of genes related to K transport and assimilation under low K at 129 DAB was greater than that at 105 DAB (Table 1). We also found that the numbers of these genes under Control treatment were greater than that under high K treatment, including cation /H^+^ antiporter, K (+) efflux antiporter, potassium channel proteins and potassium transporters. However, only the expression of *gene39320* (*AKT1*) in fruit was markedly up-regulated under high K. This data is essentially in agreement with our physiological results (Figure 1).

*AKT1* is the first K channel of the Shaker family cloned from *Arabidopsis* (Sentenac et al., 1992). It is an important inward-rectifying K^+^ channel that is involved in K^+^ uptake from the soil by the root (Hirsch et al., 1998). *AKT1* is activated through phosphorylation, which is mediated by CBL (calcineurin B-like protein)-interacting protein kinases (CIPKs) and is repressed by *AKT1*-interacting protein phosphatase 1 (AIP1)-mediated dephosphorylation (Xu et al., 2006). In *Vitis vinifera* (Cuéllar et al., 2010, 2013) and in *Populus* (Zhang et al., 2010), the CBL-CIPK network activated an *AKT1*-type channel. Fan et al. (2014) reported that increased *AKT1* activity could enhance K^+^ uptake during long-term K^+^ starvation in watermelon root. However, this was inconsistent with our results, possibly due to the difference in tissues, as our research focus on the leaves and fruit. When the K concentration increased in leaves and fruit, H^+^-ATPase activity was activated to maintain the cell membrane potential, and the increase in extracellular H^+^ might cause an increase in the plasmalemma internal and external electric potential gradient. The improvement of *AKT1* activity promoted K^+^ influx into the cell.

In addition, an outwardly rectifying K^+^ channel, SKOR, mediates the delivery of K^+^ from stelar cells to the xylem in the roots, which is a critical step in the long-distance distribution of K^+^ from roots to the upper parts of the plant (Liu et al., 2006). Shaker channel family and the HAK/KUP/KT transporter family, which comprise nine and 13 members in *A. thaliana*, respectively, and nine and 27 members in rice, play major roles in K^+^ uptake from the soil and long-distance transport in plants (Cherel et al., 2014; Yang et al., 2014; Chen et al., 2015). We found that the express levels of only four annotated *KTs* genes (*gene6330, gene14129, gene23394*, and *gene13909*) were significantly down-regulated in fruit under low K, but there were no DEGs in leaves at 129 DAB. This may be due to the incomplete pear genome annotation information, unknown DEGs were still annotated.

### K improves the sugar accumulation in fruit through mediating the genes in carbohydrate metabolic process

K^+^ is known to act as a cofactor for the activities of many enzymes in plant cells (Zörb et al., 2014). In *Arabidopsis*, rice and sugarcane, transcriptional profiling studies of genes under low K stress have reported that the genes involved in metabolic processes account for a higher percentage of genes relative to the total number of stress-response genes, and expression changes of these genes in many crucial enzymes are crucial for metabolism, such as enzymes involved in pyruvate synthesis and sugar metabolism (Armengaud et al., 2004; Ma et al., 2012; Zeng et al., 2015). Our transcriptome data found that many genes involved in metabolic processes, including starch and sucrose metabolism, glucose and mannose metabolism, and the tricarboxylic acid (TCA) cycle were expressed in pear leaves and fruit (Table S6). The sugar metabolism in fruit is particularly intense at the ripening stage, and most of the sugar is derived from photosynthetic products in leaves. The process of sugar metabolism in many studies is clear (Li et al., 2012; Cao et al., 2013). Using the transcriptome data, we analyzed the differential expression of the key genes related to sugar and acid metabolism in leaves and fruit at maturity in response to low and high K conditions (Figure S4).

Invertase (INV) is an enzyme that catalyses the hydrolysis (breakdown) of sucrose. Twenty expressed invertase genes were identified in fruit tissues of kiwifruit (Tang et al., 2016), while only 12 were found in pear (Wu et al., 2013). In this study, two *INV* genes (*gene2888* and *gene20957*) exhibited lower expression levels in leaves and fruit under low K treatment than under high K treatment. Sucrose synthase (SUS) catalyses the reversible conversion of sucrose and a nucleoside diphosphate into nucleoside diphosphate-glucose and fructose. In *Arabidopsis*, SUS is involved in the synthesis of UDP-glucose and ADP-glucose, which are involved in cellulose and starch biosynthesis, respectively (Barratt et al., 2009). Two *SUS* genes (*gene35276* and *gene39959*) were found to be significantly down-regulated in fruit at 129 DAB, and the regulation of these genes may be linked to the decrease of sucrose concentration in leaves and fruit under low K. Fructose and glucose in pear can be phosphorylated to fructose 6-phosphate or glucose 6-phosphate by fructokinase or hexokinase (FK or HK), and hexokinase (HK) phosphorylates glucose, producing glucose 6-phosphate in most organisms (Granot, 2007).

Sorbitol is the primary photosynthate, translocated carbohydrate and reserve material in woody Rosaceae species such as apple and pear (Li et al., 2016). The sorbitol concentration in leaves was higher than that in fruit, and sorbitol has been found to be synthesized by sorbitol-6-phosphate dehydrogenase (S6PDH), which was decomposed into fructose by NADP^+^-sorbitol dehydrogenase (NADP^+^-SDH) (Park et al., 2002). Our data showed that fructose concentration under low K treatment was higher than that under Control treatment in fruit, but sorbitol concentration under low K treatment was lower than that under Control treatment at 129DAB. This may be related to the up-regulation of expression of three *SDH* genes (*gene33410, gene33408*, and *gene20259*) and two *S6PDH* genes (*gene27055* and *gene27053*).

Starch biosynthesis and accumulation are also important metabolic processes in plants; they depend on *starch synthase* (SS), starch phosphorylase (SPP) and amylase (Van der Maarel et al., 2002). Potassium deficiency in plants is known to reduce starch accumulation; the activation of starch synthetase by K would support a regulatory role of this cation in the synthesis of this carbohydrate (Nitsos and Evans, 1969). In this study, K had no effect on starch metabolism in leaves, but a large number of genes related to starch metabolism were induced under low K in fruit. Nine *SS* genes (3 up-regulated and 6 down-regulated) and fifteen *SPP* genes (6 up-regulated and 9 down-regulated) were detected (Figure S4, Table S6). At 129 DAB, the glucose concentration under low K treatment was higher than that under Control treatment in fruit (Figure 2). This showed that starch was degraded to glucose by low K for TCA cycle metabolism.

Among the metabolic enzymes, pyruvate kinase acts as a central regulator of C/N metabolism (Smith et al., 2000). The activity of pyruvate kinase can be directly inhibited after long-term K-deficiency, which induces a significant reduction in the cytoplasmic pyruvate content of root cells and leads to inhibition of glycolysis and many downstream metabolic processes (such as TCA cycle) (Armengaud et al., 2009). The expression of *PFK* (ATP-dependent phosphofructokinase), *F1,6BP*(fructose 1,6-bisphosphate), *PK* (pyruvate kinase), *MDH* (malate dehydrogenase) and *PEPC* (phosphoenolpyruvate carboxylase) genes involved in acid metabolism in fruit were significantly down-regulated under low K treatment compared to Control treatment, while there were no differences in genes expression in leaves. At the same time, we found that an increase in expression of two *ME* genes *(gene5979* and *gene34528*) under low K could increase pyruvate metabolism and produce more energy to maintain cell metabolism. These results suggest that sugar and acid metabolism in leaves and fruit were weakened by low K during fruit maturation. In addition, high K increased the concentrations of sorbitol and sucrose in leaves and fruit to improve the fruit sugar levels.

### Plant hormones and ROS might have a concomitant effect in sugar accumulation in fruit

Many hormones, such as ethylene, abscisic acid, jasmonic acid, and auxin, have been implicated in several physiological responses to nutrient deficiency (Rubio et al., 2009; Schachtman, 2012). Based on the analysis of KEGG metabolic pathways, many pathways of plant hormone signal transduction were found to be enriched in leaves and fruit under low and high K at 129 DAB (Table S7, Figure S5). These eight hormone metabolic processes were affected by different K levels. Fan et al. (2014) found that DEGs related to ethylene, jasmonic acid, cytokinin, abscisic acid, and auxin were induced by low K in YS and 8,424 seedlings. The expression of genes encoding ethylene biosynthetic enzymes and regulating ethylene levels in K^+^-deprived 8424 roots increased at 120 HAT. Susawaengsup et al. (2011) found that the levels of CKs, IAA, and ABA in shoot tips of longan (*Dimocarpus longan Lour*.) were increased by potassium chlorate treatment, and dry weight (DW) amounts in treated longan shoot tips were significantly higher than those of the controls. In this study, compared to the Control treatment, the express levels of genes related to auxin transporter *AUX1* (3 out of 4 in leaves, 2 out of 3 in fruit), cytokinin transporter *CRE1* (2 out of 4 in leaves, 12 out of 14 in fruit), and brassinosteroid transporters *BAK1* (18 out of 21 in leaves, 24 out of 33 in fruit) and *BRI1* (18 out of 21 in leaves, 21 out of 26 in fruit) were significantly down-regulated under low K treatment in leaves and fruit at 129 DAB (Table S7). It has been suggested that low K inhibits IAA, CK and BR transport in leaves and fruit, to the disadvantage of growth and development of pear leaves and fruit. Auxin can stimulate ethylene production, and ethylene can affect auxin transport and accumulation (Muday et al., 2012). *ETR* (ethylene receptor) is known as the receptor gene for ethylene and regulates the *CTR* (constitutive triple response) genes under ethylene deficiencies. The regulation of *CTR* is weakened when combined with ethylene (Hua and Meyerowitz, 1998). At 129 DAB, only one *ETR* gene was found to be up-regulated and four out of six *CTR1* genes were down-regulated under low K in fruit. Two of the upstream genes, *ERF1/2*, related to fruit ripening and senescence were down-regulated under low K in fruit. These findings indicated that K promoted the conduction of plant hormones (auxin, cytokinin, brassinosteroid, and ethylene) for fruit ripening.

With extensive research in the field of plant stress biology, it is widely appreciated that many of these stresses are interrelated and produce similar secondary messenger molecules such as reactive oxygen species (ROS), calcium (Ca^2+^), and others (Shin et al., 2005). ROS are by-products of normal cell metabolism in plants, and their accumulation is damaging to cells; however, ROS also act as important signaling molecules. The balance between production and detoxification of ROS is tightly regulated by many proteins and enzymes (Sagi and Fluhr, 2006). Peroxidases are involved in oxidative cross-linking of cell wall components (e.g., lignification) and in H_2_O_2_ detoxification and are also involved in generating reactive oxygen species coupled to oxidation of hormones and defense compounds such as indole 3-acetic acid and SA (Kawano, 2003). At 129 DAB, the expression of genes related to peroxidase metabolism was found to be down-regulated under low K between leaves and fruit and only one gene (*gene38339*) was down-regulated under high K in fruit. This result indicates that the increase of K^+^ in leaves and fruit can cause excessive accumulation of oxygenated compounds such as H_2_O_2_, which will be eliminated by peroxidase when the plants are stressed. Furthermore, genes related to cytochrome P450s (18 out of 22 in leaves, 26 out of 39 in fruit) were significantly down-regulated under low K at 129 DAB. Plant cytochrome P450s participate in numerous biochemical pathways associated with plant secondary product biosynthesis (e.g., phenylpropanoids, alkaloids, lipids), growth regulation (e.g., salicylic acid, abscisic acid, gibberellins, jasmonic acid, and brassinosteroids) and resistance to toxic compounds (Cui et al., 2010; Jensen and Moller, 2010; Mizutani and Ohta, 2010).

**Figure 5:**
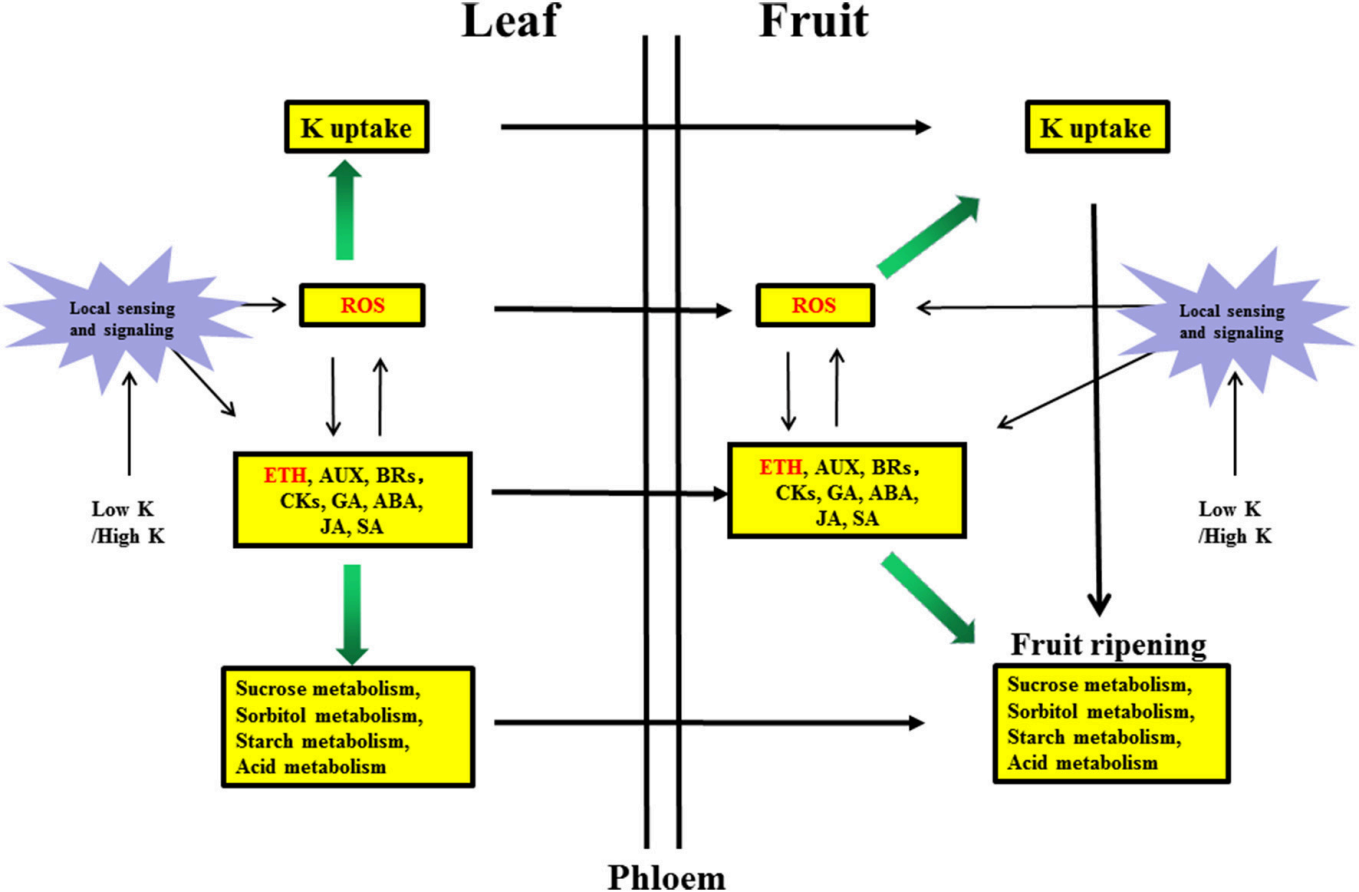
Hypothetical model of the molecular mechanism of the response in pear leaves and fruit to low K and high K. Biochemical and transport pathways are indicated with solid and dashed arrows, respectively.Purple stars represent local sensing and signaling induced by low K or high K. Yellow boxes indicate metabolic pathways responding to low K and high K in leaves and fruit. Green arrows indicate that the potassium uptake and sugar/acid metabolism were increased by hormone signaling and reactive oxygen species (ROS). ETH, Ethylene signaling; AUX, auxin signaling; BRs, brassinosteroid signaling; CKs, cytokinin signaling; GA, gibberellin signaling; ABA, abscisic acid signaling; JA, jasmonic acid signaling; SA, salicylic acid signaling.

Sugar, auxin and ROS generally accumulate in roots under nutrient deficiencies (Iqbal et al., 2013; Wang et al., 2014) and can positively interact with ethylene to regulate nutrient deficiency responses. These signals can also be transported via the phloem from roots to shoots (Zhang et al., 2014). The exact relationship between sugars and ethylene is not completely understood, but hexokinases play a critical role in mediating plant glucose and ethylene responses (Karve et al., 2012). Ethylene can stimulate ROS accumulation (Steffens, 2014), whereas ROS can stimulate ethylene production (Iqbal et al., 2013). We hypothesized that ROS may have a positive feedback effect on ethylene synthesis, leading to other physiological phenomena in responses to K stress (Figure 5).

## Conclusion

In the present study, the K levels, sugar concentration and photosynthetic characteristics in pear leaves and fruit were significantly influenced by different K levels during fruit development and maturation, especially under low-K treatment. To reveal the molecular mechanisms responsible for the differences in leaves and fruit in response to low K and high K, RNA-Seq of leaf and fruit samples taken at 105 and 129 DAB was performed to analyse gene expression patterns related to K uptake and sugar metabolism. Based on biological function analysis, most differentially expressed genes were enriched in the “metabolic pathways,” “hormonal signal transduction” and “ROS response.” Low K directly caused a decrease in the nutrition level of pear and in growth and development, and it affected the overall metabolism of leaves and fruit; this result was related to the down-regulation of the expression of potassium transporters and channels and of carbohydrate metabolism (including sucrose metabolism, sorbitol metabolism, starch metabolism and organic acid metabolism) genes. Sugar metabolism in fruit will be reduced under long-term low K, leading to decreased fruit quality. This study is the first comprehensive study using transcriptomic techniques to investigate how pear leaves and fruit respond to low and high K environments. These results will provide insights into the mechanistic regulation of some candidate genes, which can reveal the molecular mechanisms for improving fruit quality with supplemental K fertilizers.

## Data availability statement

All RNA-Seq raw data files are available from the NCBI SRA database (https://trace.ncbi.nlm.nih.gov/Traces/sra/).

Accession numbers: SRR5260714, SRR5260717, SRR5260749, SRR5260751, SRR5260753, SRR5260754, SRR5260760, SRR5260764, SRR5260820, SRR5260823, SRR5260825, SRR5260826, SRR5260827, SRR5260828, SRR5260829, SRR5260861, SRR5260863, SRR5260864, SRR5262228, SRR5262230, SRR5262231, SRR5262232, SRR5262233, SRR5262236, SRR5262240, SRR5262255, SRR5262257, SRR5262278, SRR5262279, SRR5262282, SRR5262286, SRR5262287, SRR5262290, SRR5262293, SRR5262308, SRR5262309.

## Author contributions

YX, CD, and QS conceived and designed the experiments; CS, XS, and YK performed the experiments; CS, CX, and LP analyzed the data; CS contributed reagents/materials/analysis tools; CS and JW wrote the paper.

### Conflict of interest statement

The authors declare that the research was conducted in the absence of any commercial or financial relationships that could be construed as a potential conflict of interest.

## Supplementary material

Figure S1 Growth and development of “Huangguan” pear fruit. **(A)** Stages of fruit development from flower to ripening fruit. S0: flowering (0 DAB), S1: physiological fruit dropping (20 DAB), S2: young fruit (50 DAB), S3: fruit rapid enlargement I (80 DAB), S4: fruit rapid enlargement II (105 DAB), S5: mature (129 DAB). **(B)** Changes in fruit fresh weight under different K levels. Mean of 8–10 fruits. Error bars represent SE. **(C)** Changes in fruit transverse diameter. Mean of 5–10 fruits. Error bars represent SE.

Figure S2 Effect of different potassium (K) levels on leaf photosynthetic characteristics at fruit rapid enlargement stage. Net photosynthetic rate (Pn), stomatal conductance (Gs), intercellular CO_2_ concentration (Ci), and transpiration rate (Tr) are shown. Each point represents the average of ten samples. Error bars represent SE. Statistical analysis was performed by LSD test at *P* < 0.05.

Figure S3 The qRT-PCR validation of selected 17 differentially expressed genes detected via digital transcript abundance measurements. The pear β*-TUB* gene was used as a control for normalization of expression. Development stages of pear fruit were 105 and 129 DAB. The experiment was set up using the following three K_2_O levels: 0 (K0), 0.4 (K0.4), and 0.8 (K0.8) g kg^−1^ soil, respectively. The left y-axis shows the relative gene expression levels analyzed by qPCR (black lines). The right y-axis indicates the corresponding expression data of RNA-seq (white histogram). The x-axis represents the different K levels of in leaf and fruit at 105 and 129 DAB. Bars represent SE (*n* = 3).

Figure S4 A comprehensive model of expression pattern of genes involved in sugar and main organic acid biosynthesis pathways in “Huangguan” pear leaf and fruit at 129 DAB. L1 and L2 indicate the DEGs of L0S5 vs. L0.4S5 and L0.8S5 vs. L0.4S5, F1 and F2 indicate the DEGs of F0S5 vs. F0.4S5 and F0.8S5 vs. F0.4S5. Positive fold change values (red) indicate up-regulation, whereas negative fold change values (blue). SUT, Sucrose transporter; SUS, sucrose synthase; INV, Invertase; HK, hexokinase; S6PDH, sorbitol-6-phosphate dehydrogenase; SDH, sorbitol dehydrogenase; SOT, sorbitol transporter; PGI, phosphoglucose isomerase; PFK, phosphofructokinase; F1,6BP, fructose 1,6-bisphosphate; SPP, starch phosphorylase; SS, starch synthase; PEPC, phosphoenolpyruvate carboxylase; PEPCK, phosphoenolpyruvate carboxylase kinase; PK, pyruvate kinase; MDH, malate dehydrogenase; ME, malic enzyme; Suc, sucrose; Glu, glucose; Fru, fructose; Sor, sorbitol; Star, starch; Mal, maltose; F6P, fructose 6-phosphate; G6P, glucose 6-phosphate; G1P, glucose 1-phosphate; ADPG, ADP-glucose; PYR: Pyruvate; CoA, Coenzyme A; OAA: oxaloacetate; CIT: citrate; ICIT: isocitrate; αKG: α-Ketoglutaricae; SUC: succinate; MAL: malate; TCA, tricarboxylic acid cycle.

Figure S5 DEGs were mapped to plant hormone signal transduction pathways in KEGG database in “Huangguan” pear leaf and fruit at 129 DAB. L1 and L2 indicate L0S5 vs. L0.4S5 and L0.8S5 vs. L0.4S5, F1 and F2 indicate F0S5 vs. F0.4S5 and F0.8S5 vs. F0.4S5. Positive fold change values (red) indicate the up-regulation, whereas negative fold change values (green). The number in box indicates the number of up-regulated/down-regulated genes.

Table S1 List of primer sequences used for qPCR analysis.

Table S2 Summary of the RNA-Seq data collected from the leaf and fruit of “Huangguan” pear at 105DAB and 129DAB.

Table S3 Summary of the genome alignment distribution of the RNA-Seq data.

Table S4 Summary of GO categories gene counts of the differentially expressed genes.

Table S5 Summary of KEGG categories gene counts of the differentially expressed genes.

Table S6 List of the differentially expressed genes involved in sugar and acid metabolism in response to low K and high K.

Table S7 List of the differentially expressed genes involved in plant hormone metabolism in response to low K and high K.

Table S8 List of the differentially expressed genes involved in oxidative stress in response to low K and high K.
