# Supplementary figures and images for: Transcriptome Analysis of Differentially Expressed Genes Induced by Low and High Potassium Levels Provides Insight into Fruit Sugar Metabolism of Pear
Source: Front Plant Sci. 2017 May 31;8:938. doi: 10.3389/fpls.2017.00938 (PMC5450510; doi:10.3389/fpls.2017.00938)

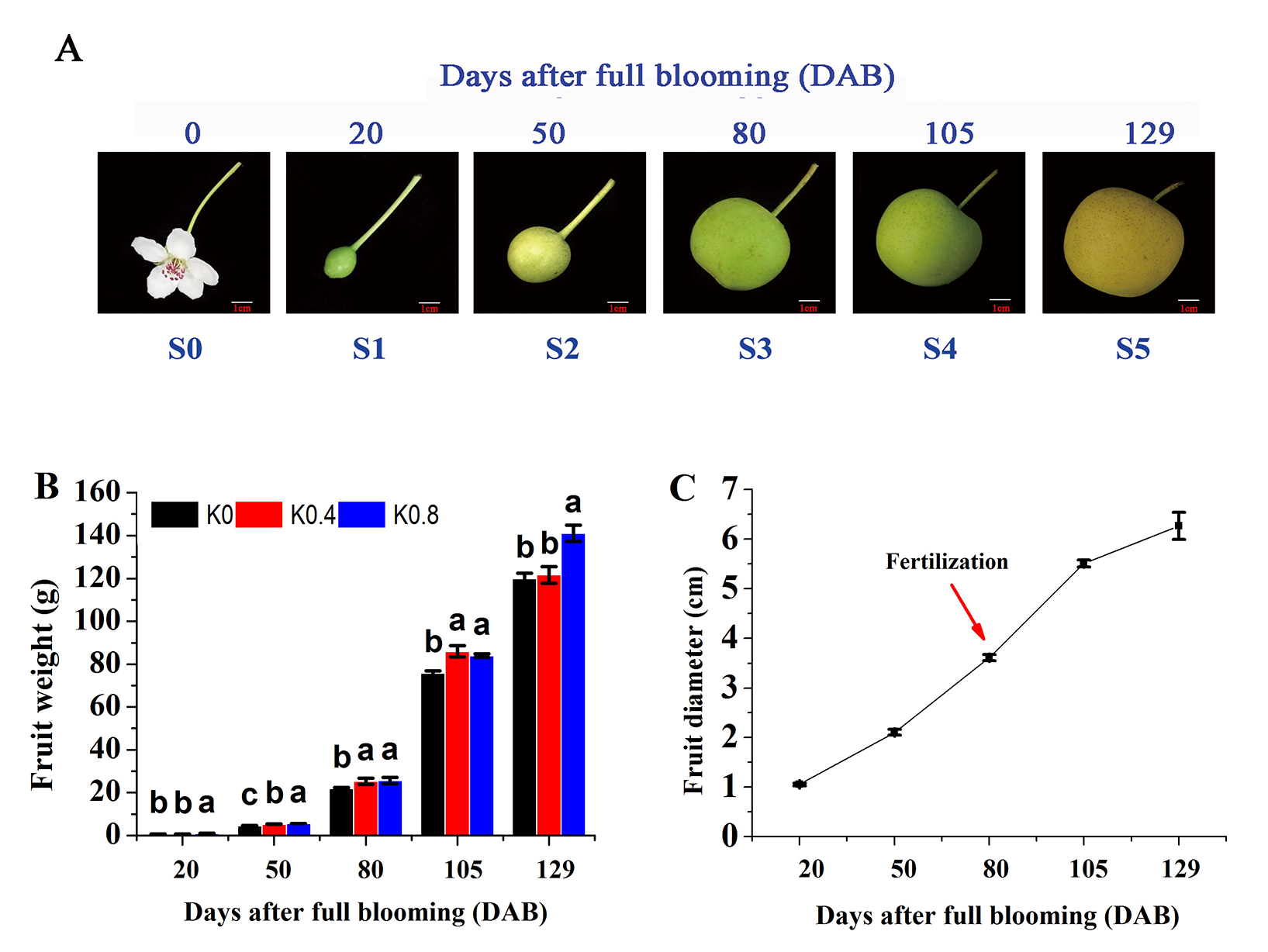

Supplement: Figure S1 — Growth and development of “Huangguan” pear fruit. (A) Stages of fruit development from flower to ripening fruit. S0: flowering (0 DAB), S1: physiological fruit dropping (20 DAB), S2: young fruit (50 DAB), S3: fruit rapid enlargement I (80 DAB), S4: fruit rapid enlargement II (105 DAB), S5: mature (129 DAB). (B) Changes in fruit fresh weight under different K levels. Mean of 8–10 fruits. Error bars represent SE. (C) Changes in fruit transverse diameter. Mean of 5–10 fruits. Error bars represent SE. [file Image1.TIF]

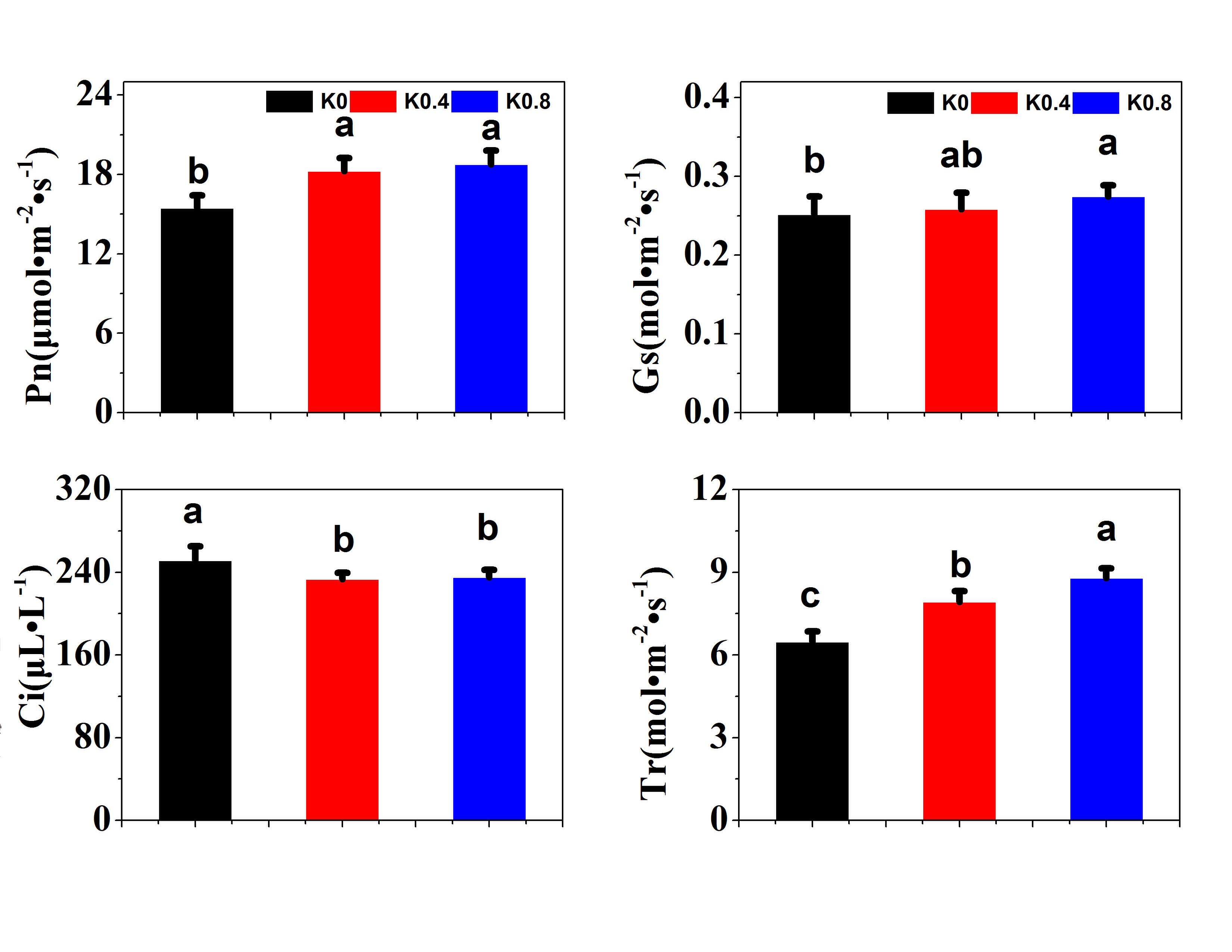

Supplement: Figure S2 — Effect of different potassium (K) levels on leaf photosynthetic characteristics at fruit rapid enlargement stage. Net photosynthetic rate (Pn), stomatal conductance (Gs), intercellular CO2 concentration (Ci), and transpiration rate (Tr) are shown. Each point represents the average of ten samples. Error bars represent SE. Statistical analysis was performed by LSD test at P < 0.05. [file Image2.TIF]

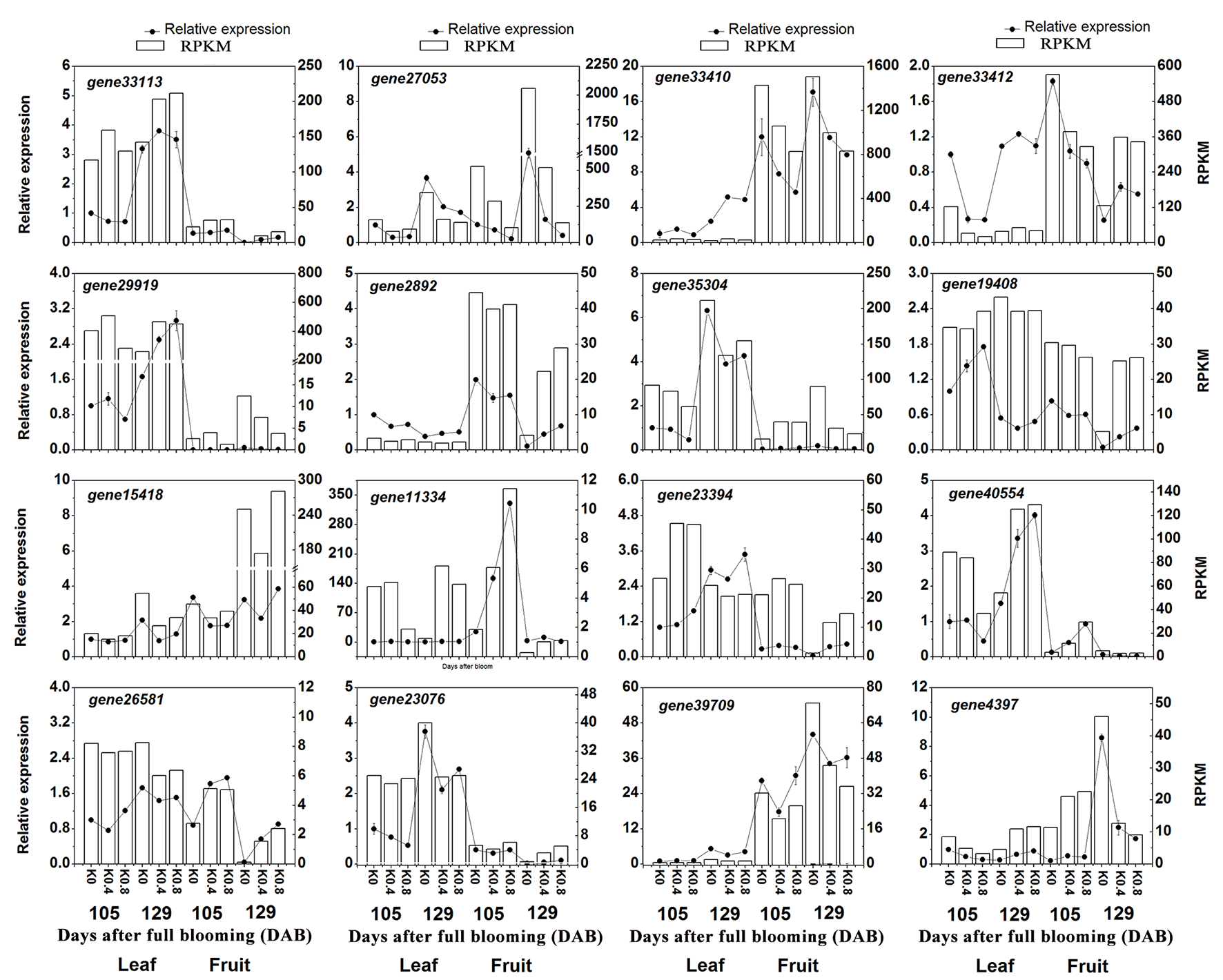

Supplement: Figure S3 — The qRT-PCR validation of selected 17 differentially expressed genes detected via digital transcript abundance measurements. The pear β-TUB gene was used as a control for normalization of expression. Development stages of pear fruit were 105 and 129 DAB. The experiment was set up using the following three K2O levels: 0 (K0), 0.4 (K0.4), and 0.8 (K0.8) g kg−1 soil, respectively. The left y-axis shows the relative gene expression levels analyzed by qPCR (black lines). The right y-axis indicates the corresponding expression data of RNA-seq (white histogram). The x-axis represents the different K levels of in leaf and fruit at 105 and 129 DAB. Bars represent SE (n = 3). [file Image3.TIF]

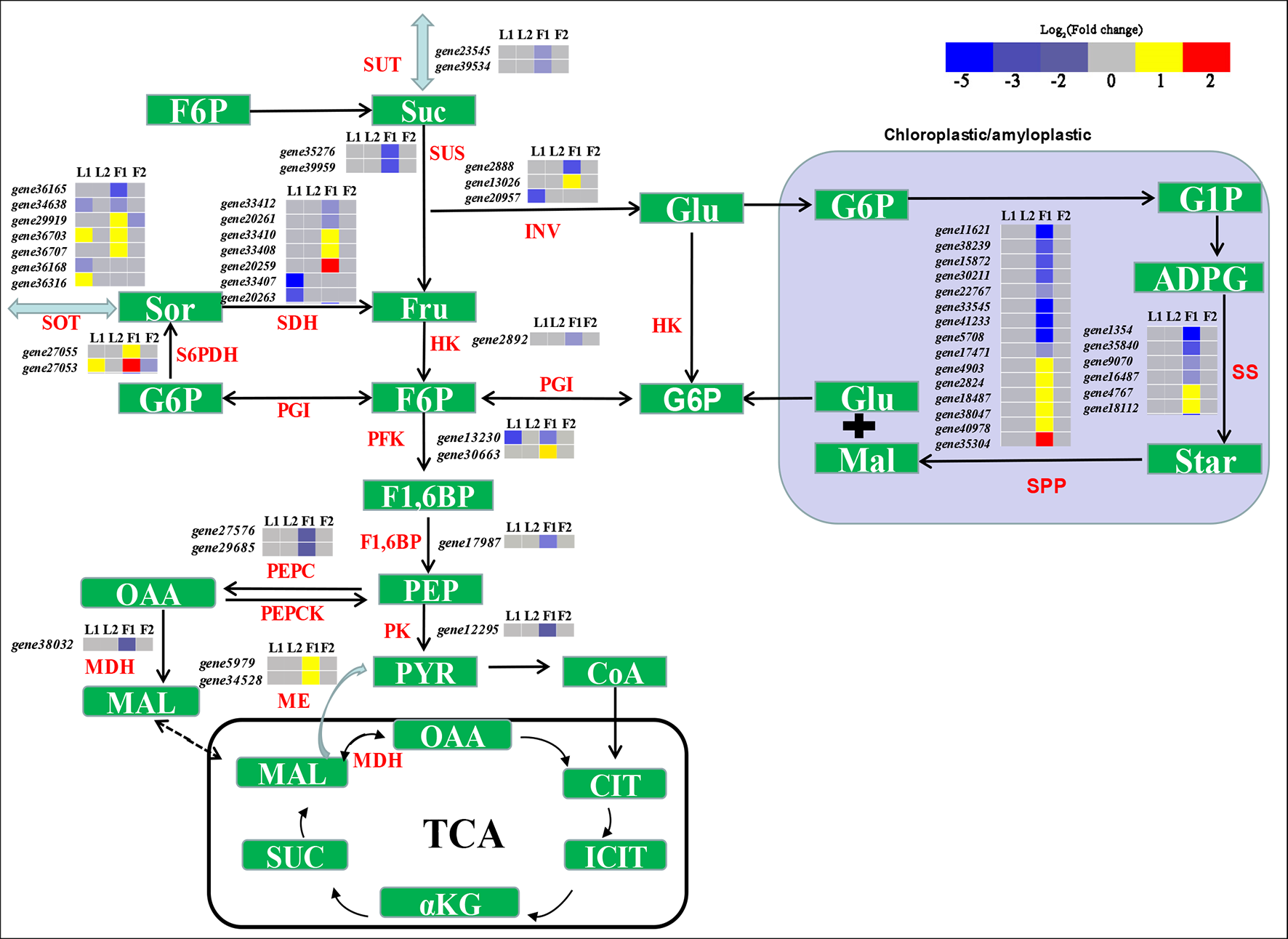

Supplement: Figure S4 — A comprehensive model of expression pattern of genes involved in sugar and main organic acid biosynthesis pathways in “Huangguan” pear leaf and fruit at 129 DAB. L1 and L2 indicate the DEGs of L0S5 vs. L0.4S5 and L0.8S5 vs. L0.4S5, F1 and F2 indicate the DEGs of F0S5 vs. F0.4S5 and F0.8S5 vs. F0.4S5. Positive fold change values (red) indicate up-regulation, whereas negative fold change values (blue). SUT, Sucrose transporter; SUS, sucrose synthase; INV, Invertase; HK, hexokinase; S6PDH, sorbitol-6-phosphate dehydrogenase; SDH, sorbitol dehydrogenase; SOT, sorbitol transporter; PGI, phosphoglucose isomerase; PFK, phosphofructokinase; F1,6BP, fructose 1,6-bisphosphate; SPP, starch phosphorylase; SS, starch synthase; PEPC, phosphoenolpyruvate carboxylase; PEPCK, phosphoenolpyruvate carboxylase kinase; PK, pyruvate kinase; MDH, malate dehydrogenase; ME, malic enzyme; Suc, sucrose; Glu, glucose; Fru, fructose; Sor, sorbitol; Star, starch; Mal, maltose; F6P, fructose 6-phosphate; G6P, glucose 6-phosphate; G1P, glucose 1-phosphate; ADPG, ADP-glucose; PYR: Pyruvate; CoA, Coenzyme A; OAA: oxaloacetate; CIT: citrate; ICIT: isocitrate; αKG: α-Ketoglutaricae; SUC: succinate; MAL: malate; TCA, tricarboxylic acid cycle. [file Image4.TIF]

PLANT HORMONE SIGNAL TRANSDUCTION

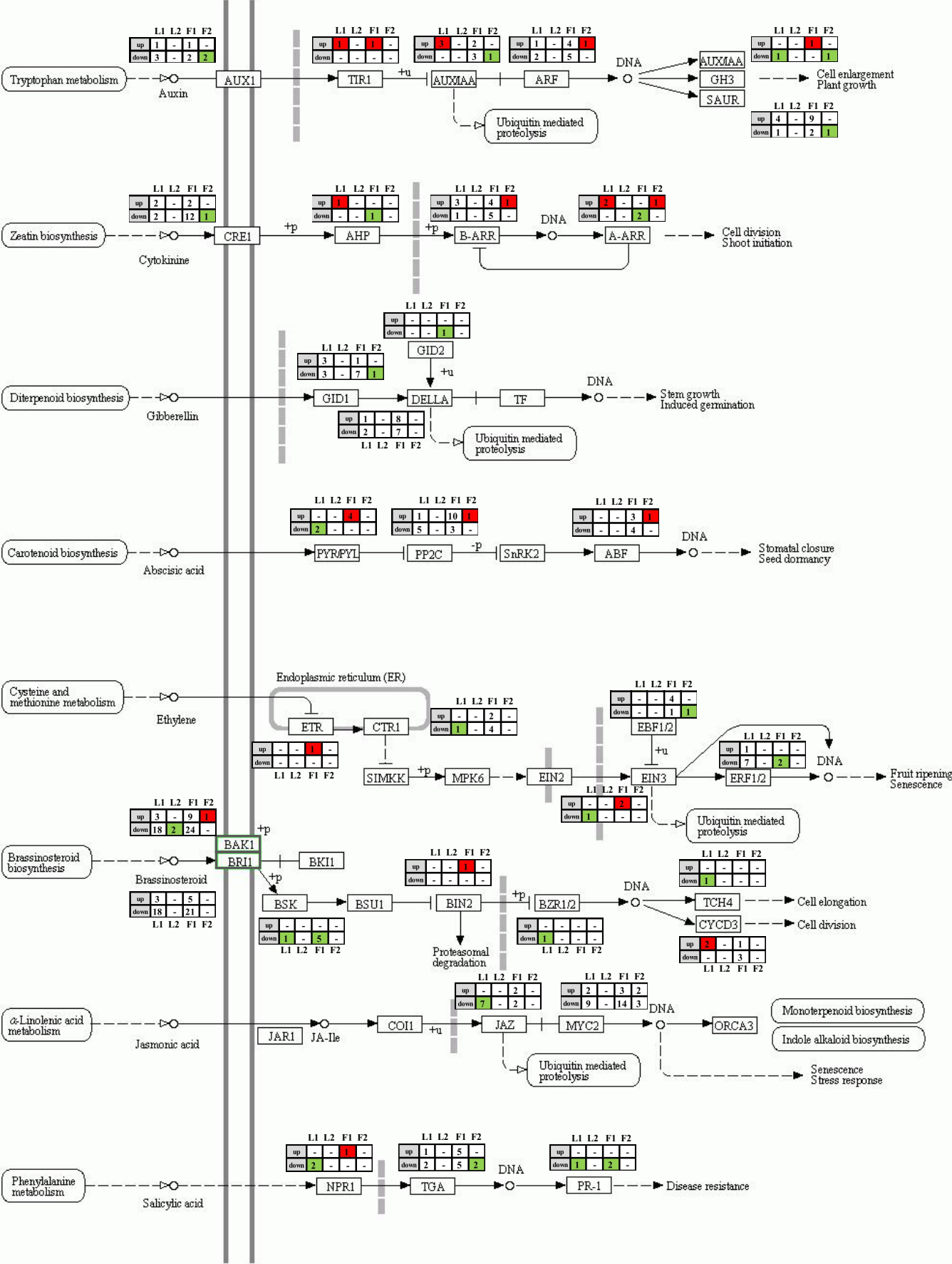

Supplement: Figure S5 — DEGs were mapped to plant hormone signal transduction pathways in KEGG database in “Huangguan” pear leaf and fruit at 129 DAB. L1 and L2 indicate L0S5 vs. L0.4S5 and L0.8S5 vs. L0.4S5, F1 and F2 indicate F0S5 vs. F0.4S5 and F0.8S5 vs. F0.4S5. Positive fold change values (red) indicate the up-regulation, whereas negative fold change values (green). The number in box indicates the number of up-regulated/down-regulated genes. [file Image5.PDF]
